# Traditional Complementary and Alternative Medicine Use Among Korean American Older Adults: A Multilevel Analysis

**DOI:** 10.1093/geroni/igaf122.1452

**Published:** 2025-12-31

**Authors:** Grace Yi, Yuri Jang

**Affiliations:** California State University, Fullerton, Fullerton, California, United States; University of Southern California, Los Angeles, California, United States

## Abstract

Korean American older adults (KAOAs) often face challenges in accessing the conventional healthcare system due to limited knowledge of the healthcare system, financial constraints, and cultural or linguistic differences that may affect their health beliefs and behaviors. As a result, many KAOAs turn to traditional complementary and alternative medicine (TCAM). However, research on TCAM use among this population remains limited. To address this knowledge gap, this study examined individual and geographic level factors associated with TCAM utilization. Data were drawn from the Study of Older Korean Americans (SOKA), a cross-sectional survey conducted between 2017 and 2018 across five states (California, New York, Texas, Hawaii, and Florida) (N = 2,150). Binomial logistic and multilevel logistic regression analyses were conducted. Findings from binomial logistic regression showed that several individual-level factors were positively associated with TCAM use, including health/well-being factors [arthritis (OR = 1.55, p<.001), health needs (OR = 1.12, p<.01), and lack of interest in work/leisure (OR = 1.74, p<.01)]; acculturation factor (use English frequently, OR = 1.45); demographic factors [gender (OR = 1.31), spirituality (OR = 1.31), perception Korean community density (OR = 1.38)]. Multilevel analysis further revealed significant state-level differences in TCAM use after controlling for the individual factors. These findings suggest that age-related physical/emotional changes may drive TCAM utilization, highlighting its potential role as a preventive health measure among ethnic minority older adults. Variations in TCAM use across states reveal the influence of community and geographic factors that shape the health-promoting behaviors of ethnic minorities, calling for culturally tailored healthcare services.

